# Psychological and Sleep Effects of Tryptophan and Magnesium-Enriched Mediterranean Diet in Women with Fibromyalgia

**DOI:** 10.3390/ijerph17072227

**Published:** 2020-03-26

**Authors:** Alejandro Martínez-Rodríguez, Jacobo Á. Rubio-Arias, Domingo J. Ramos-Campo, Cristina Reche-García, Belén Leyva-Vela, Yolanda Nadal-Nicolás

**Affiliations:** 1 Faculty of Sciences, University of Alicante, 03690 Alicante, Spain; 2 Effort Physiology Laboratory Research Group, Department of Health and Human Performance, Faculty of Physical Activity and Sport Science-INEF, Universidad Politécnica de Madrid, 28040 Madrid, Spain; jacobo.rubio@gmail.com or; 3 Faculty of Sports, Catholic University of Murcia, 30107 Murcia, Spain; djramos@ucam.edu; 4 Faculty of Nursing, Catholic University of Murcia, 30107 Murcia, Spain; creche@ucam.edu; 5 Department of Health, Vinalopó University Hospital, 03293 Elche, Spain; bmleyva@vinaloposalud.com; 6 Faculty of Medicine, Miguel Hernández University of Elche, 03202 Elche, Spain; yolanda.nadal@umh.es

**Keywords:** micronutrients, nutrition, therapy, chronic disease

## Abstract

Anxiety, mood disturbance, eating and sleep disorders, and dissatisfaction with body image are prevalent disorders in women with fibromyalgia. The authors of this study aimed to determine the effects of tryptophan (TRY) and magnesium-enriched (MG) Mediterranean diet on psychological variables (trait anxiety, mood state, eating disorders, self-image perception) and sleep quality in women with fibromyalgia (*n* = 22; 49 ± 5 years old). In this randomized, controlled trial, the participants were randomly assigned to the experimental group and the placebo group. The intervention group received a Mediterranean diet enriched with high doses of TRY and MG (60 mg of TRY and 60 mg of MG), whereas the control group received the standard Mediterranean diet. Pittsburgh Sleep Quality Questionnaire, Body Shape Questionnaire, State–Trait Anxiety Inventory (STAI), Profile of Mood States (POMS-29) Questionnaire, Eating Attitudes Test-26, and Trait Anxiety Inventory were completed before and 16 weeks after the intervention. Significant differences were observed between groups after the intervention for the mean scores of trait anxiety (*p* = 0.001), self-image perception (*p* = 0.029), mood disturbance (*p* = 0.001), and eating disorders (*p* = 0.006). This study concludes that tryptophan and magnesium-enriched Mediterranean diet reduced anxiety symptoms, mood disturbance, eating disorders, and dissatisfaction with body image but did not improve sleep quality in women with fibromyalgia.

## 1. Introduction

Fibromyalgia (FM) is defined as a heterogeneous, complex, and chronic rheumatologic syndrome characterized by musculoskeletal pain and psychological and physical exhaustion and by the fact that an individual does not show a specific disease related to similar symptoms [1,2,3]. Several studies indicate that FM is especially common in adult females aged 20 to 55 years and is also associated with several comorbidities [4]. FM affects millions of people worldwide, and its prevalence is approximately 2–3% of the abovementioned population [1,5]. Although numerous scientific studies regarding FM have been done during the last decades, the precise etiology and pathophysiology of FM are still unknown. 

Several studies about the diagnosis of FM conclude that it is a widespread condition [6]. In fact, the idea that FM is due to a multifactorial mechanism has gained much support in the last years [7]. In this sense, several complaints, individual or in combination, have been reported by FM patients. These complaints are related to exhaustion, sleep disorders, psychological disturbances in the mood state, such as anxiety or depression, self-image issues, eating disorders [1,2,5,8,9,10].

Normally, pharmacological treatments are the first-line therapy and reduce the symptoms of FM [3,11] but they are often associated with adverse effects [12]. A multidimensional approach is increasingly used as a therapy against FM symptoms. This approach is based on therapies including interventions on patient education, behavioral therapy, exercise, pain management, and relief of chronic symptoms [3]. Furthermore, some authors suggest involving diet strategies [1,13]. 

Hormonal and neurotransmission responses in FM patients showed that low serotonin levels are involved in FM. Regarding biochemical mediators, the proper intake of tryptophan (TRY), an essential amino acid precursor of serotonin [1] and one of the major contributors to the catabolic process [12], through food in a suitable diet, could improve and remit most FM symptoms (depressive disorders, mood state, pain, fatigue, and sleep disorders) [9,10,14,15,16,17,18]. Although it has not been demonstrated in humans yet, TRY supplementation also diminished hyperalgesia and reduced serum cortisol concentrations in rats [19]. Furthermore, magnesium (MG) may be involved in psychological disorders [9,13,15,20,21] and biochemical reactions, including protein synthesis and muscle and nerve function [22]. Nevertheless, while studies have shown that MG supplementation may help FM patients, evidence for other clinical outcomes remains generally weak [22]. 

Regarding the presence of these micronutrients (TRY and MG) in food, TRY is mainly contained in animal products, though a substantial content of TRY and MG is also found in some of vegetables. In this respect, nuts have high amounts of both TRY and MG [23]. Evidence suggests decreasing the ingestion of meat and animal products for the treatment of FM [14,19], as well as for the improvement of health in general. This dietary regimen corresponds with the basis and standards of the Mediterranean diet, which includes high-quality food and may also help relieve FM physiological (inflammatory), neurological, and psychological symptoms [24,25,26,27].

There are few clinical studies investigating the effects of nutritional support in FM patients undergoing regular moderate physical exercise. For this reason, the main aim of this study was to analyze the effects of tryptophan and a magnesium-enriched Mediterranean diet on psychological variables (trait anxiety, self-image issues, mood state, eating disorders) and sleep in women with fibromyalgia.

## 2. Materials and Methods

### 2.1. Trial Design

The present study employed a randomized clinical trial design to determine whether TRY and MG-enriched Mediterranean diet could improve psychological and sleep parameters in women with fibromyalgia. Randomization was carried out electronically (https://www.randomizer.org) by block design into two arms (control and experimental group) using a validated online system [28].

### 2.2. Subjects

Twenty-two women diagnosed with fibromyalgia were included in this randomized, controlled trial. Before the study, women with fibromyalgia were recruited from public fibromyalgia associations in Alicante (Spain), and all patients were interviewed and clinically evaluated in order to verify their eligibility. In the past, some of the subjects had taken oral medication, but the absence of oral medication intake was required for the subjects to be included in this study. Participants were randomly allocated into two groups. Experimental group (EG): TRY and MG-enriched Mediterranean diet (EG: Age = 48 ±4 years old and Body Mass Index (BMI) = 28.2 ± 3.7 kg/m^2^) and control group (CG: Age = 50 ± 5 years old and BMI = 28.6 ± 5.1 kg/m^2^). 

### 2.3. Declarations: Ethics Approval, Consent to Participate, and Consent for Publication

The present study was performed in accordance with the standards of Helsinki declaration. Besides, this study was approved by the Ethical Committee (UA-2019-04-10) of Alicante University, Spain. Signed informed consent was obtained from the participants. Furthermore, researchers kept confidentially all participants’ personal data, codifying the personal information for that purpose. This study was also registered as a Clinical Trial in clinicaltrial.gov (Ref. NCT04158388).

### 2.4. Eligibility Criteria

Only women were included in this study. Women aged 40–60 years with fibromyalgia, officially diagnosed based on the American College of Rheumatology fibromyalgia diagnosis criteria in 2016 [29], were included in the study.

Participants who agreed to be included in the trial were requested not to make any changes to their lifestyle and follow the suggested dietary pattern during the intervention program. Exclusion criteria from the study included: use of analgesics, vitamins-containing supplements, and other drugs for the treatment of fibromyalgia symptoms and participation in other clinical trials.

### 2.5. Intervention Protocol

The intervention was performed for 16 weeks. During the intervention program, all the participants were instructed to follow a prescribed isocaloric diet plan based on the Mediterranean diet (only with food, no supplements). Both groups’ diet was based on this macronutrient distribution: 55% carbohydrate (mainly complex carbohydrates), 15% protein, and 30% fat (control group: mainly from olive oil; experimental group: from walnuts). Calorie intake average was 1700 ± 300 Kcal; macronutrient and distribution regarding body weight was: 3.5 ± 0.6 g of carbohydrate/kg body weight; 0.9 ± 0.2 g of proteins/kg body weight; 1.9 ± 0.2 g of fats/kg body weight for both groups. Control and experimental groups’ diet included 350 mg of TRY and 375 mg of MG (≈5 mg/kg body weight), following standardized recommendations (RDA) established by the National Academy of Sciences [30]. Furthermore, the experimental group received a Mediterranean diet enriched with a higher dose of TRY and MG (60 mg of TRY and 60 mg of MG) derived from eating walnuts at both breakfast and dinner (3–5 units), since walnuts contain with high levels of TRY and MG (0,17g of TRY and 158 mg of MG/100g of walnuts) [31]. 

To ensure and check the full compliance of the Mediterranean diet and the TRY and MG-enriched Mediterranean diet, participants were met weekly and contacted by phone twice per week by a dietitian. 

### 2.6. Measurements

Body composition was assessed at the baseline to describe the sample. Body weight and height, using a digital scale (TANITA, Tokyo, Japan) and stadiometer (SECA, Hamburg, Germany), were measured, following the International Society for the Advancement of Kinanthropometry (ISAK) guidelines, to calculate the BMI as “weight(kg)/height^2^ (m)”. 

Data collection was performed on 2 different days. All the psychological and sleep inventories or questionnaires were collected at the baseline and after three months of intervention. 

Sleep quality was assessed using the Pittsburgh Sleep Quality Index (PSQI) [32], validated in Spanish [33]. The PSQI is a self-reported questionnaire that evaluates sleep quality and disturbance in the preceding month and contains 19 entries on seven dimensions (subjective sleep quality, sleep latency, sleep duration, habitual sleep efficiency, sleep disturbances, use of sleeping medication, and daytime dysfunction, all measured in the range 0–3). The sum of the components produces a global score ranging from 0 to 21; higher scores indicate worse sleep quality. A total score greater than 5 indicates that the individual presents major dysfunctions in at least two components or moderate dysfunction in at least three components. 

Our clinical assessment included the Spanish version of the Body Shape Questionnaire (BSQ) [34] developed by Cooper et al. (1987). The BSQ allows the specific valuation of the worry linked to the perception of one’s body image and comprises 34 items that assess dissatisfaction with one’s own body-shape. The items are answered on a 6-point Likert scale (1 never, 6 always). The clinical cut-off score for the Spanish population is 105 (see [34]). 

Also, the State–Trait Anxiety Inventory (STAI) [35] in Spanish [36] was used only for the trait state. It consists of an auto-administered test of 20 items evaluating each item from 0 to 3 on the Likert scale (0 = almost never, 1 = sometimes, 2 = often, 3 = almost always). It measures Trait Anxiety (TA) as anxiety tendency, is relatively stable over time, and can be considered as providing a general tendency of an individual’s anxiety. The total score is obtained by summing the values of the items (after reversing the negative scores), and the higher the score is, the greater the anxiety. Mild anxiety is considered between 20 and 25 points, moderate anxiety between 26 and 32, and high anxiety 33 or more points. 

Mood and mood changes were assessed with the abbreviated Spanish version of the Profile of Mood States (POMS-29) [37], developed by McNair, Lorr, and Droppleman (1971), that consists of 29 self-rated adjectives on a five-point scale from 0 (not at all) to 4 (extremely). The scale reports on five moods: tension (reflects an increase in musculoskeletal tension), anger (shows a mood of anger and dislike for others), vigor (represents a high-energy state), fatigue (represents a low-energy state), and depression (reflects a low mood or a depressed mood). Total mood disturbance (TMD) is derived from POMS using the following formula, TMD = (sum of all subscales except vigor) – vigor. 

Finally, the risk of developing eating disorders (eating disorders) was evaluated using the Eating Attitudes Test-26 (EAT-26) [38]. It was validated in the Spanish population from 12 years of age [39]. It consists of 26 items that make up three subscales: dieting (e.g., I avoid foods with sugar in them), bulimia and food preoccupation (e.g., I vomit after I have eaten), and oral control (e.g., I cut my food into small pieces). Participants provided their answers on a 6-point Likert-type scale, from never to always. A score of ≥20 or a “yes” answer to five behavioral questions was considered indicative of disordered eating behavior. 

### 2.7. Statistical Analysis

Statistical analysis of data was performed with SPSS v.24 (IBM, Armonk, NY, USA) in a Windows environment. Descriptive statistics with measures of central tendency and dispersion were used. The assumption of normality and homoscedasticity was verified with the Shapiro–Wilk Test and the Levene’s test. Finally, homogeneity of variance–covariance matrices assumption was evaluated with the Box’s M test. A two-way (group × moment) analysis of variance with repeated measures and Bonferroni post-hoc test were used to investigate training effects and differences between groups when a significant interaction was observed. The effect size was calculated using ETA squared (η²). For all procedures, the level of significance was set at *p* ≤ 0.05 [40]. 

## 3. Results

**Figure 1:**
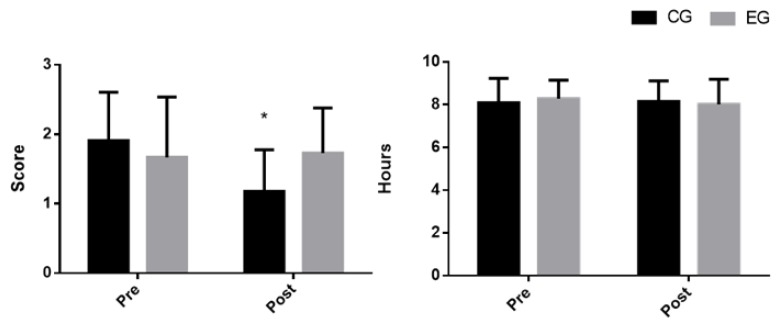
Effects of tryptophan and magnesium-enriched Mediterranean diet on (a) sleep duration rating (left) and (b) sleep duration (h) (right); *: *p* value < 0.01; CG: control group; EG: experimental group.

Table 1 shows the sleep quality variables measured by the Pittsburg questionnaire. A significant main effect of group x time was observed in sleep duration rating (F = 7.62; *p* = 0.013; η² = 0.061), with a significant decrease in CG (*p* < 0.001) at the end of the treatment (Figure 1). In addition, there was a main effect of group x time on sleep duration (F = 4.57; *p* = 0.047; η² = 0.019). No other main effect of group x time was observed in sleep quality variables. However, there was a main effect of time on sleep efficiency (F = 9.3; *p* = 0.007; η² = 0.059).

After statistical analysis, no main effect of group x time was found in BSQ. However, there was a main effect of time on bulimia (F = 5.53; *p* = 0.029; η² = 0.017). Results from STAI questionnaire comparison between CG and EG showed a main effect of time on trait anxiety (F = 14.5; *p* = 0.001; η² = 0.181). Concerning the POMS changes investigated, no main effect of group x time was observed in any variable measured (Table 2). In addition, no main effect of time was observed on anger, vigor, and tension. There was a main effect of time on depression (F = 18.63; *p* < 0.001; η² = 0.062), fatigue (F = 12.36; *p* = 0.002; η² = 0.073), and the overall results of POMS (F = 14.03; *p* = 0.001; η² = 0.105).

Regarding the eating disorders changes analyzed, no main effect of group x time was observed in any variable measured (Table 2). However, there was a main effect of time on diet (F = 10.61; *p* = 0.004; η² = 0.025). In addition, no main effect of time was observed on bulimia or oral control. 

## 4. Discussion

The aim of this study was to analyze the effects of the Mediterranean diet enriched with tryptophan and magnesium on psychological variables (anxiety trait, self-image perception, mood, eating disorders) and on sleep quality in women with fibromyalgia.

According to Bjørklund et al. (2018) [9], diet changes regarding some nutrients may help reduce pain in fibromyalgia patients. In addition, TRY is an essential amino acid and a precursor of the neurotransmitter serotonin. Tryptophan metabolites, such as serotonin and melatonin, are thought to participate in the regulation of mood and sleep, and tryptophan is used to treat insomnia, sleep apnea, and depression [41]. In addition, TRY bioavailability could affect the inflammatory system [42]. On the other hand, MG deficiency has been related to pain increment in fibromyalgia patients [9], and the ageing process affects MG absorption [43]. In addition, a low MG intake has been associated with poor-quality sleep and inflammatory stress [44]. 

In the present study, we observed that a Mediterranean diet enriched with tryptophan and magnesium produced some psychological benefits in women with fibromyalgia. Our results showed lower anxiety scores after the intervention, characterized by a lower tendency to perceive situations as threatening, less emotional instability, reduced feelings of sadness, loneliness, and fear. In addition, we observed a mood disturbance decrease in the EG, who showed low levels of fatigue (represents a low-energy state) and depression (reflects a low mood or a depressed mood). 

Recent studies made in healthy middle-aged women revealed that a daily consumption of a low-dose supplement containing bioavailable tryptophan may have beneficial effects on emotional and cognitive functions [10]. It is known that there are biological factors (such as tryptophan—a building block of serotonin—depletion) which strongly influence the appearance of depressive disorders [45]. Tryptophan hydroxylase-2 (*TPH-2*) gene may play an important role in maintaining the normal level of serotonin in the central nervous system [46,47], and, as suggested by Ping et al. (2019) [17], neurotransmission by serotonin in the brain may be associated with changes in white matter integrity in patients with major depressive disorder. On the other hand, we know that tryptophan intake is inversely associated with the level of self-reported depression [41] and also affects the inflammatory system [42]. 

Furthermore, disturbed moods and anxiety in people with fibromyalgia may be associated with dysfunctional eating habits [48]. In the case of our patients, we observed that the EG after the intervention showed less unhealthy diet-related behaviors, which could indicate effects on eating behavior disorders, or as Alberdi-Páramo and Niell-Galmés [49] suggested, a precipitation of tryptophan deficits. 

Initially, the participants of our study did not report concern and dissatisfaction about their body image in relation to body weight and shape. After the intervention, lower scores appeared for both CG and EG groups.

Sleep efficiency, and actual sleep time in the presence of sleep difficulties, was improved in middle-aged/elderly people by the intake of identical doses of tryptophan to those used in the present study [50]. This optimization of sleep may be due to an improvement of the function of the inflammatory system and to better values in self-reported levels of depression and anxiety [42]. In addition, Samad et al. [51] found that TRY supplementation can help subjects with sleep disturbances and may be positively linked with sleep duration [30,41]. Futhermore, Silber and Schmitt [6] observed that TRY loading appears to be most effective in improving mood in vulnerable subjects and sleep in adults with some sleep disturbances. However, we observed no changes in sleep disruptions in the population of our study after the intervention. On the other hand, the sleep duration values decreased significantly in the CG, whereas but no change was observed in the EG. These findings are in accordance with previous reports, which observed no improvements in sleep quality after TRY [52,53] ingestion and no associations between dietary MG consumption and sleepiness [54] Thus, these controversial finding, that the participants did not improve their sleep quality, could be related to the dosage of TRY and MG micronutrients. Therefore, the RDA established by the National Academy of Sciences [30] and the higher doses of TRY and MG (60 mg of TRY and 60 mg of MG per day) previously recommended [31] were not enough to improve sleep quality in women with fibromyalgia. In addition, the plasma concentrations of TRY and MG were not assessed, and this may affect the sleep hygiene response of the participants. 

From an application perspective, physicians and dietitians who treat women with fibromyalgia should keep in mind that the administration of a Mediterranean diet enriched with 60 mg of TRY and 60 mg of MG improves some symptoms linked with fibromyalgia, such as anxiety, mood disturbance, eating disorders, dissatisfaction with body image and sleep quality. 

The main limitation of the present study is that the participants that took part in the study were only middle-aged women, who are more likely to be susceptible to dietary TRY manipulation than men [6,55]. Inclusion of only middle-aged female participants could limit the external validity of the study. The number of participants should also be increased in further studies, because few participants were included in this study. In addition, our results cannot be generalized to other subjects who are in another range of age (i.e., older women); neither these findings can be generalized to and across other conditions or diseases. Even though we requested the participants not to make any change to their lifestyle, in particular in exercise habits as a potential important confounding variable, in future studies, authors should consider tracking how much exercise the patients perform during the study. Furthermore, it could be useful to record life stressors for the study participants to discriminate whether or not observed differences are due to or influenced by changes in stress levels, considering that stress contributes to pain amplification.

In terms of the methodological procedures employed herein, women with fibromyalgia were psychological assessed subjectively by a self-report for the assessment of eating disorders, not through an accurate interview. 

In addition, the results of plasma TRY and MG concentration were not determined in blood parameters in this study. Moreover, the fact that some physiological (i.e., actigraphy or electroencephalography measures of sleep quality instead of questionnaires) and plasma concentration variables were not assessed may also be considered a potential limitation. 

The main strengths of the present study are its novelty and the practical application of the results to the clinical field. Although 16 weeks should be a sufficient amount of time to begin seeing changes in the experimental group, no differences were observed in the present study. Dietitians supervised dietary prescription and intake registration for all the participants three times per week, to be sure the participants were following the close diet prescription. Nevertheless, offering both groups pre-prepared meals with accurately studied nutritional contents could be another possibly strategy to minimize the possibility of confounding variables linked with a diet prescription, even if it is supervised by dietitians. Besides, in future studies, the specific effects of micronutrients should be studied by administering them separately from food, because the effects of individual micronutrients vary when they are administered with food which contains them at low levels and not in pure form.

Further research on the influence of micronutrients supplementation on fibromyalgia symptoms is necessary. Therefore, our observations should firstly be confirmed in other studies with women with fibromyalgia and further explored using prospective studies. Furthermore, the generalized pain positivity, widespread pain, symptom severity, and polysymptomatic distress scales should be used to evaluate the baseline characteristics of the study group participants, in order to better understand the spectrum of severity of FM. 

## 5. Conclusions

Daily consumption of a Mediterranean-diet enriched with a high dose of TRY and MG (60 mg of TRY and 60 mg of MG) by middle-aged women with fibromyalgia during 16 weeks had modest beneficial effects on emotional processing, decreased fatigue, anxiety, and depression, and reduced possible eating disorders and dissatisfaction with body image, but did not modify sleep quality.

## Figures and Tables

**Table 1:** Effects of tryptophan and magnesium-enriched Mediterranean diet on the Pittsburgh Sleep Quality Index (PSQI).

Group	*n*	Mean	SD	Mean Post	SD Post	Time			Time × Group		
						F	*p*	ES η²	F	*p*	ES η²
PITTSBURG											
Subjective sleep quality rating											
Control	11	1.55	1.51	1.18	1.33	1.67	0.211	0.013	0.034	0.856	0
Experimental	11	1.55	1.44	1.27	1.49						
Latency											
Control	11	1.55	0.93	1.55	0.82	0.132	0.721	0	0.132	0.721	0
Experimental	11	2	1	2.09	1.04						
Sleep Duration rating											
Control	11	1.91	0.7	1.18	0.6	7.62	0.013	0.061	7.62	0.013	0.061
Experimental	11	1.67	0.87	1.73	0.65						
Sleep Duration (h)											
Control	11	8.09	1.14	8.15	0.96	2.81	0.111	0.012	4.57	0.047	0.019
Experimental	11	8.28	0.87	8.01	1.18						
Sleep efficiency, %											
Control	11	73.65	16.21	82.43	12.58	9.3	0.007	0.059	0.57	0.461	0.004
Experimental	11	71.7	17.55	75.27	11.44						
Habitual sleep efficiency rating											
Control	11	1.36	1.29	0.91	1.04	4.35	0.052	0.034	0.1	0.752	0
Experimental	11	1.56	1.01	1.36	1.03						
Mean sleep disturbances rating											
Control	11	1.73	0.65	0.91	0.47	0.000	1.000	0.000	0.000	1.000	0.000
Experimental	11	1.91	0.3	1.91	0.54						
Mean sleep medication rating											
Control	11	0.55	0.82	0.27	0.65	3.5	0.076	0.059	0.07	0.792	0.001
Experimental	11	0.64	0.5	0.27	0.65						
Mean daytime dysfunction rating											
Control	11	1.82	0.75	1	0	7.62	0.019	0.272	0.85	0.377	0.03
Experimental	11	1.91	0.54	1.38	0.74						
Global sleep quality index											
Control	11	10.18	3.63	7.55	2.46	5.15	0.034	0.042	3.4	0.08	0.028
Experimental	11	10.27	4.13	10	3.55						

*n*: sample number; SD: standard deviation; Post: post-intervention; F: F-statistic; *p*: *p* value; ES: effect size; η²: ETA squared.

**Table 2:** Body Shape Questionnaire (BSQ), State–Trait Anxiety Inventory (STAI), Profile of Mood States (POMS), and Eating Attitudes Test-26 (EAT-26) results for control and experimental groups.

Group	*n*	Mean	SD	Mean Post	SD Post	Time			Time × Group		
						F	*p*	ES η²	F	*p*	ES η²
**BSQ**											
Control	11	66.64	39.09	60.00	41.05	5.53	**0.029**	0.017	0.37	0.550	0.001
Experimental	11	54.09	32.10	42.82	23.65						
**STAI**											
**Trait Anxiety**											
Control	11	9.73	3.55	5.55	2.58	14.5	**0.001**	0.181	0.13	0.721	0.001
Experimental	11	11.55	5.84	8.09	3.70						
**POMS Depression**											
Control	11	7.64	4.20	5.09	4.93	18.63	**<0.001**	0.062	0.88	0.359	0.003
Experimental	11	4.73	3.50	3.09	3.27						
**POMS Anger**											
Control	11	11.82	5.98	7.73	4.10	2.58	0.124	0.0435	0.866	0.363	0.001
Experimental	11	11.82	6.06	10.73	8.24						
**POMS Vigor**											
Control	11	4.91	2.17	5.18	4.83	0.003	0.959	0	0.1315	0.721	0.000
Experimental	11	10.09	5.30	9.73	4.43						
**POMS Fatigue**											
Control	11	13.64	3.01	10.18	4.47	12.36	0.002	0.073	2.63	0.120	0.016
Experimental	11	11.18	4.51	9.91	5.01						
**POMS Tension**											
Control	11	36.45	8.64	38.27	10.45	0.365	0.553	0.003	2.467	0.132	0.024
Experimental	11	31.64	7.32	27.55	9.52						
**Total Score**											
Control	11	137.91	13.15	123.36	12.15	14.03	**0.001**	0.105	1.66	0.212	0.013
Experimental	11	129.18	19.13	122.09	19.10						
**Eating Disorders (EAT-26)**											
**Diet**											
Control	11	12.55	8.29	10.18	9.61	10.61	**0.004**	0.025	0.054	0.818	0.000
Experimental	11	7.00	6.83	4.27	5.85						
**Bulimia**											
Control	11	5.00	4.54	5.27	3.10	0.045	0.833	0	0.045	0.883	0.000
Experimental	11	3.91	2.51	3.91	2.66						
**Oral control**											
Control	11	4.36	3.23	3.73	3.41	1.107	0.305	0.012	0.005	0.945	0.000
Experimental	11	3.00	3.19	2.27	2.45						
**Total Score**											
Control	11	21.91	13.25	17.73	15.01	9.273	**0.006**	0.063	0.01	0.919	0.000
Experimental	11	13.91	10.38	10.00	9.47						

*n*: sample number; SD: Standard deviation.
